# Maternal hyperhomocysteinemia induces fetal growth restriction by suppressing angiogenesis at the maternal-fetal interface

**DOI:** 10.1186/s13578-025-01529-0

**Published:** 2026-01-09

**Authors:** Sujuan Li, Yichi Wu, Yuan Gao, Anran Tian, Minglan Yao, Fucheng Meng, Furong Liang, Yingying Li, Cai Zhang, Xiaoping Luo

**Affiliations:** 1 Department of Pediatrics, Tongji Hospital, Tongji Medical College, Huazhong University of Science and Technology, 1095 Jiefang Avenue, Wuhan, 430030 People’s Republic of China; 2 Hubei Provincial Key Laboratory of Pediatric Genetic Metabolic and Endocrine Rare Diseases, Wuhan, 430030 People’s Republic of China; 3 Hubei Provincial Clinical Research Center for Children’s Growth and Development and Metabolic Diseases, Wuhan, 430030 People’s Republic of China; 4 State Key Laboratory for Diagnosis and Treatment of Severe Zoonotic Infectious Disease, Wuhan, 430030 People’s Republic of China

**Keywords:** Fetal growth retardation, Hyperhomocysteinemia, Maternal-fetal interface, Angiogenesis, Lipid metabolism

## Abstract

Maternal hyperhomocysteinemia (HHcy) is closely linked to fetal growth restriction (FGR), yet the underlying mechanisms remain incompletely understood. In this study, we established a rat model of HHcy by administering a high-methionine diet during pregnancy and confirmed the presence of FGR through fetal weight analysis. Histological evaluation of the maternal-fetal interface revealed reduced vascular density in both the decidua and placenta, accompanied by dysregulated expression of key angiogenic factors in decidua. To elucidate the mechanistic basis of these changes, primary decidual stromal cells (DSCs) were isolated and RNA sequencing was performed. HHcy impaired the proangiogenic capacity of DSCs by suppressing vascular endothelial growth factor A (VEGFA) secretion. Transcriptomic profiling identified significant enrichment of lipid metabolism pathways in HHcy-exposed decidua. Further molecular analyses revealed that CD36 played a central role in mediating HHcy-induced lipid metabolic disturbances, which in turn activated the peroxisome proliferator-activated receptor (PPAR) signaling pathway. Importantly, pharmacological inhibition of CD36 in DSCs alleviated lipid accumulation, suppressed PPAR pathway activation, and restored VEGFA expression and secretion, thereby rescuing DSCs-mediated angiogenesis. Collectively, our findings suggest that maternal HHcy upregulates CD36 expression in DSCs, leading to lipid metabolism dysregulation and impaired VEGFA-mediated angiogenesis possibly via the PPAR pathway, ultimately contributing to the pathogenesis of FGR. This is the first study to implicate lipid metabolism as a critical regulator of decidual angiogenesis, offering novel mechanistic insights and a potential therapeutic target for HHcy-associated pregnancy complications.

**Supplementary Information:**

The online version contains supplementary material available at 10.1186/s13578-025-01529-0.

## Introduction

Fetal growth restriction (FGR) is a pathological condition in which the fetus fails to achieve its genetic growth potential during intrauterine development [1]. Globally, the prevalence of FGR is estimated at 17.4%, with a reported rate of approximately 6.4% in China [2, 3]. As a common pregnancy complication, FGR is associated not only with neonatal morbidity and mortality, but also with an elevated risk of long-term metabolic disorders in adulthood, including obesity, type 2 diabetes, and cardiovascular disease [4, 5]. Despite its clinical importance, the pathogenesis of FGR remains incompletely understood, and effective preventive or therapeutic interventions are currently lacking.

Homocysteine (Hcy) is a sulfur-containing, non-essential amino acid produced as an intermediate in the methionine cycle [6]. Its metabolism is influenced by both genetic and nutritional factors, including polymorphisms in enzymes involved in one-carbon metabolism and dietary intake of methionine, folate, betaine, vitamin B6, and vitamin B12 [7]. Disruption of these pathways results in elevated plasma Hcy levels, a condition known as hyperhomocysteinemia (HHcy). In China, the prevalence of HHcy in adult women is approximately 10%, with notable regional variation-ranging from 17% in northern regions to 2.6% in the south [8]. HHcy has been implicated in systemic vascular dysfunction and is associated with conditions such as hypertension, cardiovascular disease, and neurodegenerative disorders [9]. Notably, emerging evidence links HHcy to FGR, but the underlying mechanisms remain poorly defined [10–13].

The pathogenesis of FGR is multifactorial, involving maternal, fetal, and maternal-fetal interface components [1]. The decidua in early pregnancy and the placenta in later stages together constitute the maternal-fetal interface, where angiogenesis is vital to fetal growth [14, 15]. The vascular development at this interface is tightly regulated by angiogenic factors, among which vascular endothelial growth factor A (VEGFA) and placental growth factor (PLGF) play pivotal roles [16, 17]. In early pregnancy, the decidua not only supports implantation and fetal growth but also governs placental vascular remodeling [18]. Decidual stromal cells (DSCs), the predominant cell type within the decidua, as a major source of VEGFA, play critical roles in promoting placental angiogenesis and vascular remodeling [19]. Nevertheless, the effects of HHcy on DSCs’ function remain unexplored.

Previous studies have shown that HHcy impairs angiogenesis, largely through its deleterious effects on endothelial cells [20]. Although some evidence suggests that HHcy may inhibit extraembryonic vascular development by downregulating angiogenic mediators, its specific role in regulating angiogenesis at the maternal-fetal interface, particularly through effects on DSCs, remains unknown [21]. To better understand the mechanisms underlying HHcy-induced FGR, we used a rat model of gestational HHcy to assess its effects on fetal and placental development, maternal-fetal interface angiogenesis, and the functional impact of HHcy on DSC-mediated VEGFA secretion and angiogenesis.

## Materials and methods

### Animals

Eight-week-old Sprague-Dawley rats were obtained from Beijing Vital River Laboratory Animal Technology Co., Ltd (Beijing, China). Female and male rats were mated together overnight at a ratio of 2:1. The presence of vaginal sperm plugs was designated as embryonic day (E) 0. Pregnant dams were individually housed and randomly assigned to two groups based on the diet throughout gestation: the normal diet (ND) group received standard chow (methionine content: 0.78%), while the high methionine diet (HMD) group was provided with a diet containing 2% methionine to induce maternal HHcy by referencing the modeling method of MILYUTINA et al. (Jiangsu Medicience Biopharmaceutical Co., Ltd, Jiangsu, China) [22].

Pregnant rats were anesthetized via intraperitoneal injection of 2% pentobarbital sodium at E10, E14, and E20, respectively. Embryos/fetuses, decidua, placentas, and maternal serum were collected via cesarean section. All rats were housed in the SPF animal experiment center of Tongji Hospital. Animal procedures were conducted in accordance with the Chinese guidelines and regulations for experimental animals, and approved by the Animal Care and Use Committee of Tongji Hospital (Approval No.TJH-202109011).

### UHPLC-MS

Serum metabolites involved in the Met cycle were quantified by UHPLC-MS as previously described with slight modifications [23]. Stock solutions (50 mmol/L) of Hcy (44925, Supelco, USA), Met (PHR1241, Supelco, USA), S-adenosylmethionine (SAM, A4377, Sigma, USA), and S-adenosyl-L-homocysteine (SAH, A9384, Sigma, USA) were prepared in methanol: water (85:15, v/v). The working solutions and quality control solutions were obtained by diluting stock solutions in acetonitrile: water (85:15, v/v) containing internal standard (IS, D3-Met, 250 nmol/L, 300616, Sigma, USA). For serum sample preparation, 200 µL of methanol with 0.5% formic acid and IS (312.5 nmol/L) was added to 50 µL of serum for protein precipitation. After vortexing for 30 s and centrifugation at 15,000 g for 10 min at 4 ℃, the supernatant was subjected to UHPLC-MS analysis using an Xevo TQ-S Triple Quadrupole Mass Spectrometer (Waters Corporation, USA) with an ACQUITY UPLC BEH Amide column (2.1 × 100 mm, 1.7 μm, Waters Corporation, USA).

The mobile phases were water (A) and acetonitrile (B), both containing 0.1% formic acid. The LC gradient was: 0 min, 85% B; 9 min, 45% B; 10 min, 30% B; 11 min, 85% B; 14 min, 85% B. The injection volume was 10 µL. Data were acquired and processed using Thermo Xcalibur software (version 4.1, Thermo Scientific, USA).

### Immunofluorescence staining

Tissues were fixed in 4% paraformaldehyde, dehydrated in gradient ethanol, embedded in paraffin, and sectioned at 6 μm. After deparaffinization, rehydration, and antigen retrieval with boiling citrate buffer under pressure for 2 min, sections were blocked with 5% goat serum and incubated overnight at 4 °C with CD31 antibody (1:2000, Abcam, ab182981). Secondary antibody (1:5000, Abclonal, China) incubation was followed for 2 h at room temperature. Nuclei were counterstained with DAPI (Servicebio, Wuhan, China), and images were captured using a fluorescence microscope (IX71, OLYMPUS, Tokyo, Japan). Angiogenesis was quantified in at least four tissues per group across five visual fields per sample using AngioTool software [24].

### Western blot

Proteins from tissues or cells were extracted using RIPA buffer (BOSTER, Wuhan, China) and quantified by BCA assay (BOSTER). Samples were separated by SDS-PAGE and transferred to nitrocellulose membranes, blocked in 5% skim milk for 2 h, and incubated with primary antibodies (Supplementary Table S1) overnight at 4 °C. Membranes were then incubated with secondary antibodies and visualized with a chemiluminescence system (ChemiDoc XRS+, Bio-Rad, California, USA). Band intensities were analyzed with Image Lab software and normalized to GAPDH or Vinculin.

### Quantitative real-time PCR

Total RNA was extracted from tissues or cells using RNAiso plus (Takara, Tokyo, Japan) and cDNA was synthesized using PrimeScript RT Master Mix (Takara, Tokyo, Japan) according to the manufacturer’s protocol. All amplifications were performed on CFX Connect Real-Time PCR Detection System (Bio-Rad, Hercules, CA, USA) using a 10 µL reaction consisting of 0.2 µL forward primer, 0.2 µL reverse primer, 1 µL cDNA, 3.6 µL H_2_O, and 5 µL SYBR Premix EX Taq (Takara, Tokyo, Japan). Expression levels were normalized to GAPDH and analyzed by the 2^−ΔΔCT^ method. The primer sequences for RT-qPCR are listed in Supplementary Table S2.

### Extraction and culture of DSCs

DSCs were isolated from E10 decidua using a modified version of Du’s protocol [25]. Dissected decidua was digested with 0.1% type IV collagenase (Sigma, C5138, USA) containing 0.03% DNase I (Sigma, DN25, USA) at 37 °C for 1 h. The digestion was terminated with DMEM/F12 containing 10% FBS, and the solution was filtered through 100, 70, and 40 μm filters, then centrifuged at 1000 rpm for 5 min. Cells were resuspended in PBS and purified by discontinuous Percoll gradient centrifugation, yielding DSCs with 98% purity.

DSCs were cultured in DMEM/F12 supplemented with 20% FBS and 1% penicillin-streptomycin at 37 °C in a humidified 5% CO2 incubator. When 70% confluent, cells were treated with Hcy (Sigma, H4628, USA) and/or Sulfo-N-succinimidyl oleate sodium (SSO, MCE, HY-112847 A, China) for 24 h, and collected for analysis or further cultured for 24 h to obtain conditioned medium (CM) after changing to serum-free medium.

### Human umbilical vein endothelial cell culture and treatment

The immortalized human umbilical vein endothelial cells (HUVECs) were purchased from Shanghai Zhongqiao Xinzhou Biotechnology Co., Ltd (Shanghai, China). HUVECs were cultured in endothelial cell medium with 20% FBS and 1% penicillin-streptomycin at 37 °C in a humidified 5% CO2 incubator. HUVECs were plated in 24-well plates for tube formation assays or 6-well plates for wound healing experiments.

### Cell viability assay

Cell viability was measured using the Cell Counting Kit-8 (CCK8, Abbkine, China). DSCs were seeded in 96-well plates, treated with Hcy and/or SSO for 24 h, and incubated with CCK8 reagent (1:10 dilution) for 30 min–2 h. Absorbance at 450 nm was measured. Cell viability was calculated as: Cell viability (%) = (OD_Sample_ − OD_Blank_) / (OD _Control_ − OD_Blank_) × 100%.

### ELISA

VEGFA concentration in the CM of DSCs was measured using a rat VEGFA ELISA kit (Cusabio, CSB-E04757r, Wuhan, China) following the manufacturer’s instructions.

### Tube formation assay

HUVECs were seeded on Matrigel-coated 24-well plates (Corning, 356234, USA) and incubated with DSCs-CM for 6 h. Tube formation was imaged microscopically. The number of branch points and total tube length were quantified using ImageJ software.

### Wound healing experiment

HUVECs were cultured in 6-well plates until confluent, then scratched with 200 µL tips. Detached cells were washed off with PBS. After incubation in DSCs-CM for 12 h, scratch areas at 0 h and 12 h were photographed and measured with ImageJ software to calculate the cell migration ability: wound healing rate (%) = (0 h area − 12 h area) / 0 h area × 100%.

### RNA sequencing

RNA sequencing was conducted by Shanghai Majorbio Bio-pharm Technology Co., Ltd (Shanghai, China). Total RNA was extracted using TRIzol and quality was assessed with the Agilent Bioanalyzer 4150. After poly(A) + selection, RNA was fragmented and reverse-transcribed. Libraries were constructed and sequenced on the Illumina NovaSeq 6000 platform. Raw reads were processed using Fastp, aligned to the reference genome using HISAT2, quantified with StringTie, and analyzed for differential expression with DESeq2. Functional enrichment analyses were performed for differentially expressed genes (DEGs).

### Total cholesterol and triglyceride testing

Levels of total cholesterol (TC) and triglycerides (TG) in serum, decidua, and DSCs were measured using assay kits (Nanjing Jiancheng Bioengineering Institute, Nanjing, China) according to the manufacturer’s protocols.

### Statistical analysis

The quantitative data were expressed as mean ± SEM and analyzed using GraphPad Prism 8.0. Normality and homogeneity of variance were tested before applying parametric tests. Unpaired Student’s t-test was used for comparisons between two groups, and one-way ANOVA for multiple-group comparisons. The differences were considered statistically significant at *P < 0.05, **P < 0.01, ***P < 0.001, and ****P < 0.0001.

## Results

### Maternal HHcy impairs fetal growth and placental development

**Fig. 1:**
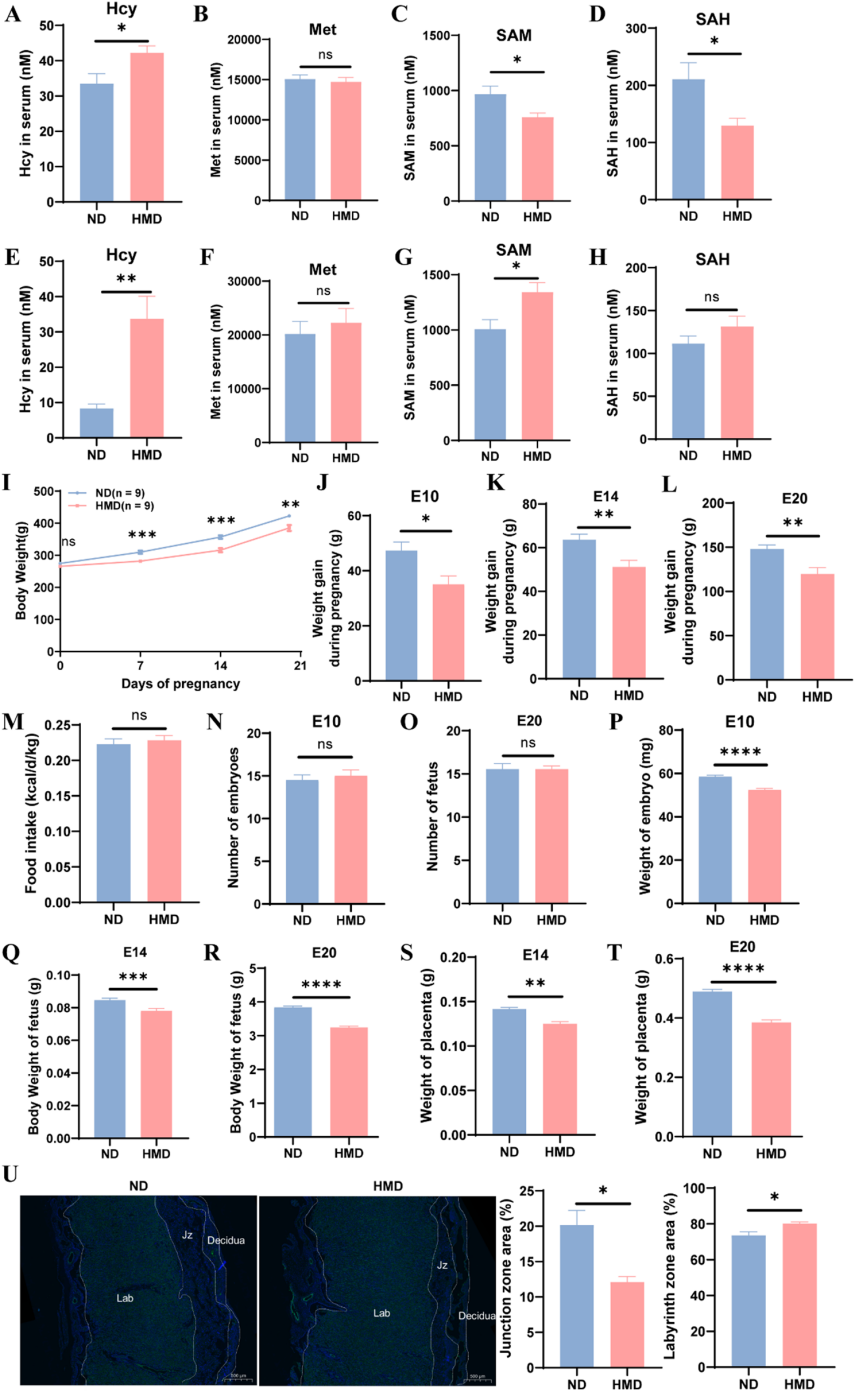
Maternal HHcy impairs fetal growth and placental development.** A**–**D** Serum levels of methionine metabolism-related metabolites at E10 (*n* = 12 per group). **E**–**H** Serum levels of methionine metabolism-related metabolites at E20 (*n* = 10 per group). **I** Gestational weight curves in ND and HMD dams (*n* = 9 per group). **J**–**L** Gestational weight gain in two groups of pregnant dams at E10 (*n* = 10 per group), E14 (*n* = 9 per group), and E20 (*n* = 9 per group). **M** Caloric intake during pregnancy in ND and HMD groups (*n* = 9 per group). **N**–**O** Number of embryos per litter in two groups at E10 (*n* = 10 per group) and E20 (*n* = 9 per group). **P** Embryo weight at E10 (*n* = 123:127). **Q** Fetal weight at E14 (*n* = 110:117). **R** Body weight of male fetuses at E20 (*n* = 85:66). **S**–**T** Placental weight at E14 (*n* = 110:117) and E20 (*n* = 85:66). **U** Representative images of CD31 immunofluorescence staining and quantitative analysis of placental structure at E20 (*n* = 4 per group). Lab: labyrinth zone; Jz: junction zone. Scale bar = 500 μm. The data were expressed as mean ± SEM and statistical comparisons were assessed by unpaired two-tailed Student’s t-test. ns *P* > 0.05, * *P* < 0.05, ** *P* < 0.01, *** *P* < 0.001, **** *P* < 0.0001

To establish an animal model of HHcy during pregnancy, SD rats were fed HMD throughout pregnancy. Maternal serum metabolites related to methionine metabolism (including Met, Hcy, SAM, and SAH) were analyzed. At E10, the serum Hcy of dams in HMD group was elevated, while the serum Met levels remained unchanged, and the serum levels of SAM and SAH decreased (Fig. 1A–D). By E20, serum Hcy increased more markedly, SAM levels showed a mild increase, and Met and SAH levels remained stable (Fig. 1E–H). These data confirmed the successful induction of HHcy during pregnancy, with more pronounced effects observed at later gestational stages.

Monitoring the pregnancy weight of dams revealed no initial differences between groups at E0. However, from E7 onward, HMD dams exhibited significantly lower body weights compared to ND dams (Fig. 1I). The weight gain during pregnancy was decreased in HMD group compared to ND group at E10, E14, and E20 (Fig. 1J–L). Moreover, no significant difference was observed in calorie intake between the two groups, indicating that impaired weight gain was not due to reduced food consumption (Fig. 1M).

At E10 and E14, although the number of embryos per litter was unchanged, the embryonic weight was significantly lower in HMD group (Fig. 1N–Q). At E20, when the fetal gender could be determined, only male fetuses and their associated placentas were included in further analysis to reduce variability and simplify the experimental design. At this stage, male fetuses in HMD group also showed significantly reduced body weight compared to ND group (Fig. 1R).

To explore potential mechanisms underlying FGR, placental development was evaluated. Placental weights were significantly lower in HMD group at both E14 and E20 (Fig. 1S and T). CD31 (the specific marker for vascular endothelial cells) immunofluorescence staining of E20 placentas revealed clear structural organization, with identifiable labyrinthine (vascular-rich), junctional (cell-dense), and decidual (vessel-containing) zones (Fig. 1U). Notably, the relative area of the junctional zone was reduced in HMD group, while the labyrinthine zone proportion was slightly increased (Fig. 1U). These findings suggest that maternal HHcy disrupts placental structure and development, potentially contributing to FGR by impairing the maternal-fetal interface.

### HHcy impairs vascular density at the maternal-fetal interface and affects angiogenic factor expression in decidua

**Fig. 2:**
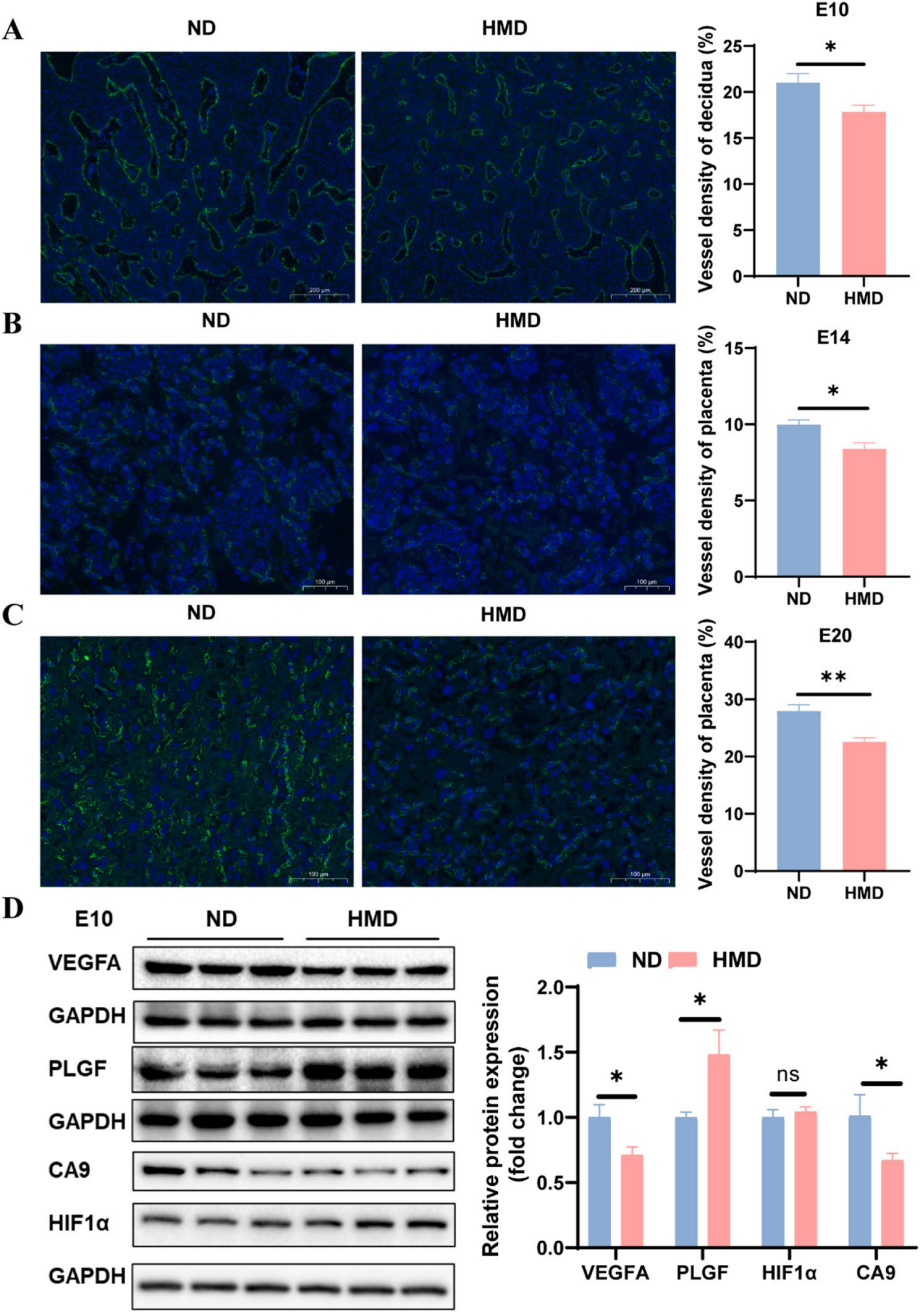
HHcy impairs vascular density at the maternal-fetal interface and affects angiogenic factor expression in decidua. **A** Representative images and quantification of CD31 immunofluorescence staining in decidua of ND and HMD pregnant rats at E10 (*n* = 4 per group). Scale bar = 200 μm. **B** Representative images and expression of CD31 immunofluorescence staining in placentas at E14 (*n* = 4 per group). Scale bar = 100 μm. **C** Representative images and expression of CD31 immunofluorescence staining in placentas at E20 (*n* = 4 per group). Scale bar = 100 μm. **D** Protein expression levels of VEGFA, PLGF, HIF1α, and CA9 in decidua at E10 (*n* = 6 per group). The data were expressed as mean ± SEM and statistical comparisons were assessed by unpaired two-tailed Student’s t-test. ns *P* > 0.05, * *P* < 0.05, ** *P* < 0.01

To elucidate the mechanisms by which maternal HHcy contributes to FGR, we assessed angiogenesis at the maternal-fetal interface by measuring vascular density across gestational stages. The decidua in early pregnancy (E10) and the placenta in later stages (E14, E20) together constitute the maternal-fetal interface, where angiogenesis is vital to fetal growth. At E10, CD31 immunofluorescence staining of decidua showed significantly reduced vascular density in HMD group (Fig. 2A). At E14, when placenta becomes the main organ supporting fetal development, CD31 staining of the placenta revealed sparse vascular networks in the labyrinthine layer, with significantly reduced vascular density in HMD group compared to ND group (Fig. 2B). By E20, placental vascular architecture had matured, yet vascular density in the labyrinthine layer remained markedly lower in HMD group (Fig. 2C).

Western blot was performed to explore the impact of HHcy on angiogenic factors expression in decidua. The results revealed that VEGFA expression was downregulated, while PLGF expression was upregulated in HMD group (Fig. 2D). Additionally, the expression of hypoxia-related marker carbonic anhydrase IX (CA9) was decreased, potentially reflecting an early compensatory response to impaired vascularization (Fig. 2D).

Collectively, these results suggest that maternal HHcy affects the expression of angiogenic factors in decidua, leading to impaired vascular development at the maternal-fetal interface throughout gestation.

### HHcy inhibits angiogenesis by reducing VEGFA secretion from DSCs

To determine whether HHcy impairs angiogenesis at the maternal-fetal interface by affecting decidual stromal cells (DSCs), primary DSCs were isolated and treated with culture media containing homocysteine (HHcy group) or control media (NC group) for 24 h.

**Fig. 3:**
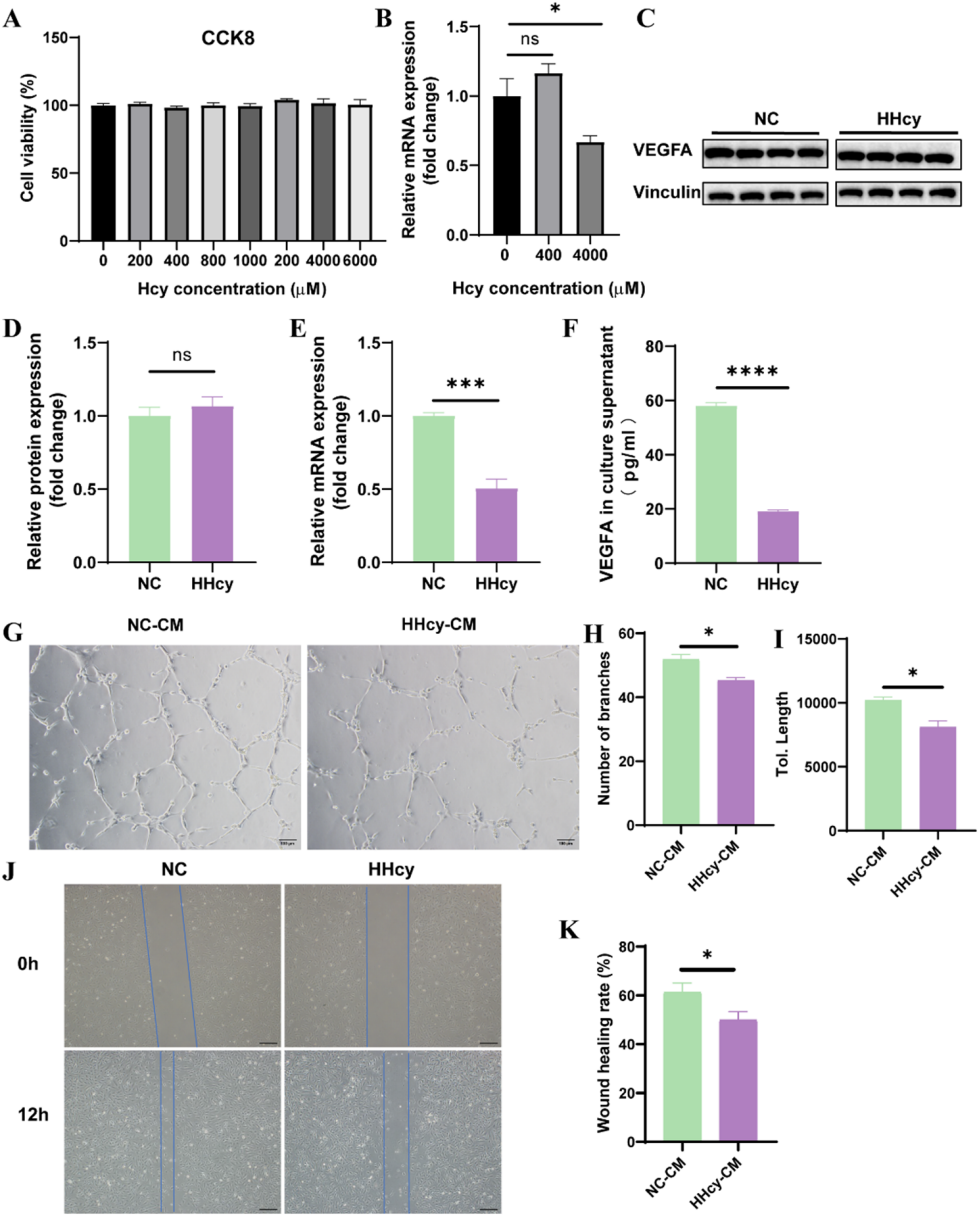
HHcy inhibits angiogenesis by reducing VEGFA secretion from DSCs. **A** Cell viability of DSCs assessed by CCK8 assay after treatment with different concentrations of Hcy for 24 h (*n* = 6 per group). **B** VEGFA mRNA expression in DSCs following treatment with varying concentrations of Hcy for 24 h (*n* = 4 per group). **C** and **D** VEGFA protein levels in DSCs treated with normal culture medium (NC) or medium containing 4000 µM Hcy (HHcy) for 24 h (*n* = 4 per group). **E** VEGFA mRNA levels in NC and HHcy groups (*n* = 4 per group). **F** VEGFA concentration in DSCs culture supernatants, analyzed by ELISA (*n* = 6 per group). **G**–**I** CM from DSCs was used for HUVECs tube formation assays. Images were taken 6 h post-seeding. Scale bar = 100 μm. Quantitative analysis of tube branch number and total tube length (*n* = 3 per group). **J** and **K** CM from DSCs was used for HUVECs wound healing assays. Scratch images were taken at 0 and 12 h. Scale bar = 50 μm. Wound healing rates were quantified (*n* = 3 per group). The data were expressed as mean ± SEM and statistical comparisons were assessed by unpaired two-tailed Student’s t-test. ns *P* > 0.05, * *P* < 0.05, *** *P* < 0.001, **** *P* < 0.0001

First, CCK8 assays revealed no significant differences in DSC viability after treatment with Hcy at concentrations ranging from 200 to 6000 µM (Fig. 3A). VEGFA mRNA expression was significantly downregulated at 4000 µM Hcy, while 400 µM had no effect (Fig. 3B). Thus, 4000 µM Hcy was chosen for subsequent experiments.

Although VEGFA mRNA expression was reduced following 4000 µM Hcy treatment, intracellular VEGFA protein levels in DSCs remained unchanged (Fig. 3C–E). Considering that VEGFA exerts its pro-angiogenic function primarily through secretion into the extracellular environment, we next quantified VEGFA levels in the cell culture supernatants. After 24 h of Hcy treatment, the culture medium was replaced with serum-free medium for an additional 24 h to allow for VEGFA secretion, and the supernatants were collected for analysis. Notably, VEGFA secretion into the extracellular space was significantly reduced in HHcy group (Fig. 3F). In summary, Hcy treatment did not alter intracellular VEGFA protein levels in DSCs, as determined by Western blotting. However, the amount of VEGFA secreted into the extracellular space, assessed by ELISA, was markedly reduced, accompanied by a significant decrease in VEGFA mRNA expression. These findings suggest that Hcy may concurrently impair both the transcriptional regulation and the secretory process of VEGFA.

To assess the functional impact of reduced VEGFA secretion on angiogenesis, CM from treated DSCs was applied to HUVECs in the tube formation assay. Compared to CM from NC group (NC-CM), CM from HHcy group (HHcy-CM) significantly impaired tube formation, as evidenced by fewer branch points and shorter total tube length (Fig. 3G–I). In the wound healing assay, HUVECs exposed to HHcy-CM also exhibited reduced migratory ability compared to NC-CM group (Fig. 3J–K).

These findings demonstrate that HHcy reduces VEGFA mRNA expression and secretion from DSCs without affecting their viability, leading to impaired angiogenesis and endothelial cell migration, which may contribute to reduced angiogenesis at the maternal-fetal interface.

### HHcy activates lipid metabolism pathways in decidua

**Fig. 4:**
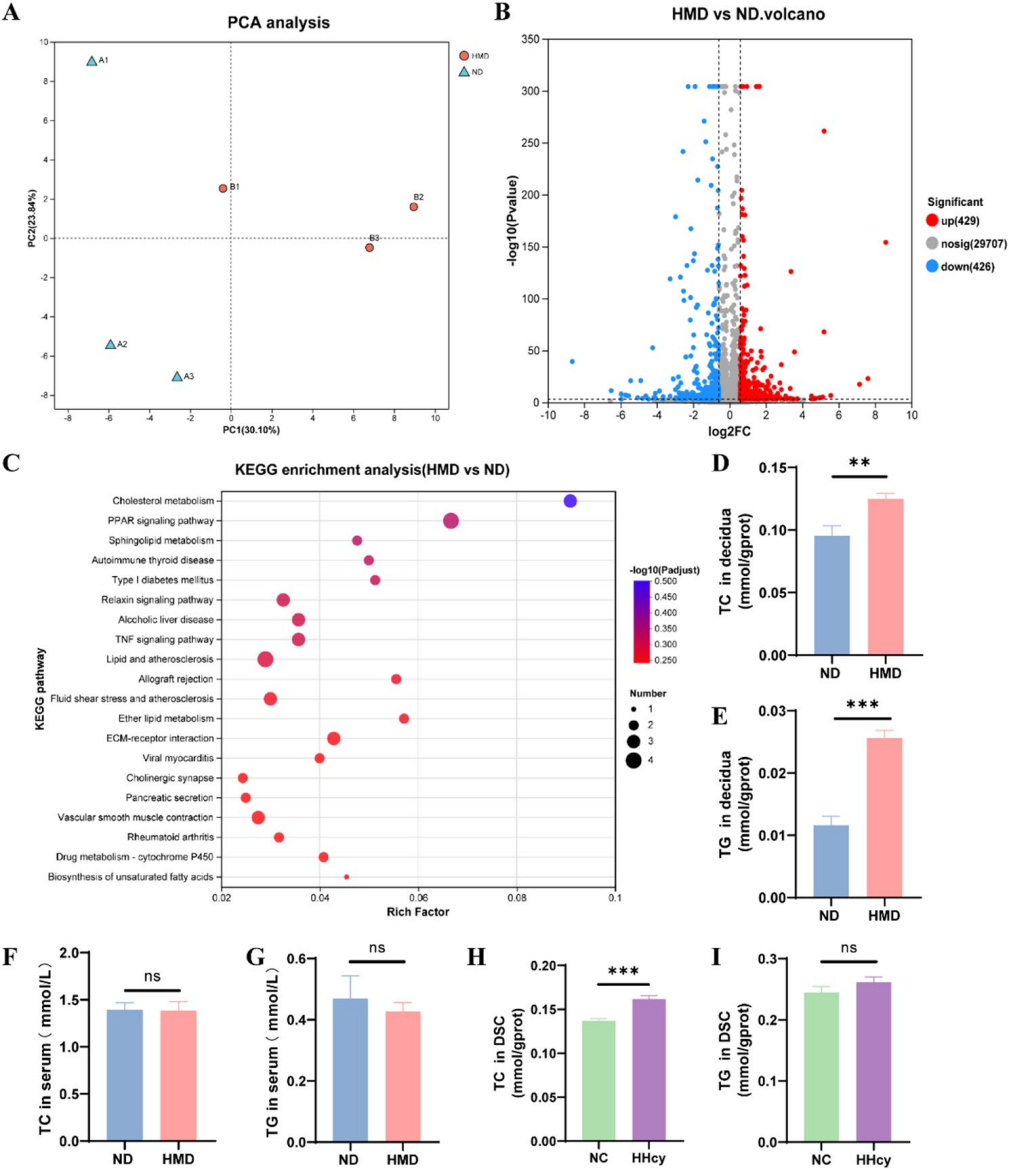
HHcy enriches the lipid metabolism pathways in decidua. **A** Principal component analysis (PCA) of gene expression profiles in decidua from ND and HMD rats. **B** Volcano plot displaying log₂ fold changes in mRNA expression in decidua. Red and blue dots indicate significantly upregulated and downregulated differentially expressed genes (DEGs), respectively (fold change > 1.5, adjusted *P* value < 0.05). **(C)** KEGG pathway enrichment analysis of DEGs in decidua. **D** and **E** TC and TG levels in decidua from ND and HMD rats (*n* = 8 per group). **F** and **G** TC and TG levels in serum from ND and HMD rats (*n* = 12 per group). **H** and **I** TC and TG levels in DSCs from NC and HHcy groups (*n* = 6 per group). The data were expressed as mean ± SEM and statistical comparisons were assessed by unpaired two-tailed Student’s t-test. ns *P* > 0.05, ** *P* < 0.01, *** *P* < 0.001

To explore the possible mechanism by which HHcy impairs the proangiogenic function of DSC, we performed RNA sequencing of E10 decidua. Principal component analysis (PCA) revealed distinct clustering between the HMD and ND groups, indicating divergent gene expression profiles (Fig. 4A). DESeq2 differential expression analysis identified 855 DEGs, with 429 upregulated and 426 downregulated in HMD group (Fig. 4B). The KEGG pathway enrichment analysis of DEGs showed a trend of enrichment in lipid metabolism pathways, including cholesterol metabolism, PPAR signaling pathway, sphingolipid metabolism, ether lipid metabolism, and biosynthesis of unsaturated fatty acids (Fig. 4C).

We assessed lipid levels in decidua and isolated DSCs to validate these findings. TC and TG levels were significantly elevated in the decidua of HMD group compared to ND group (Fig. 4D and E). Notably, serum TC and TG levels showed no significant difference between the two groups, suggesting that the increased lipid accumulation in decidua was not due to systemic hyperlipidemia (Fig. 4F and G). Lipid analysis in DSCs revealed a significant increase in TC levels under HHcy conditions, while TG levels remained unchanged (Fig. 4H and I).

These results suggest that HHcy promotes the activation of lipid metabolism pathways and induces lipid accumulation in decidua.

### CD36 is a key mediator of HHcy-induced lipid deposition in decidua

**Fig. 5:**
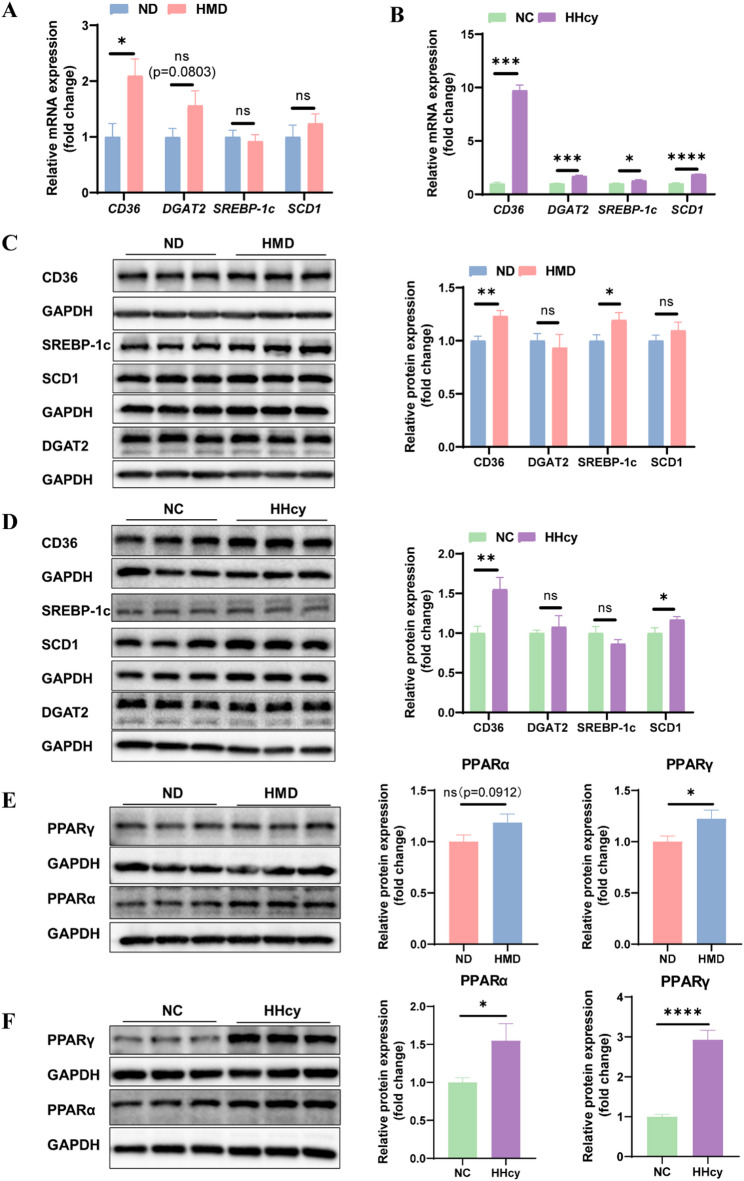
CD36 is a key mediator of HHcy-induced lipid metabolic alterations in decidua. **A** The mRNA expression levels of lipid metabolism-related genes in decidua from ND and HMD rats (*n* = 8 per group). **B** The mRNA expression levels of lipid metabolism-related genes in DSCs from NC and HHcy groups (*n* = 4 per group). **C** The protein expression levels of lipid metabolism-related molecules in decidua from ND and HMD rats (*n* = 12 per group). **D** The protein expression levels of lipid metabolism-related molecules in DSCs from NC and HHcy groups (*n* = 6 per group). **E** The protein expression levels of PPARα and PPARγ in decidua from ND and HMD rats (*n* = 12 per group). **F** The protein expression levels of PPARα and PPARγ in DSCs from NC and HHcy groups (*n* = 6 per group). The data were expressed as mean ± SEM and statistical comparisons were assessed by unpaired two-tailed Student’s t-test. ns *P* > 0.05, * *P* < 0.05, ** *P* < 0.01, *** *P* < 0.001, **** *P* < 0.0001

To identify the key regulators involved in HHcy-induced lipid metabolic alterations, we examined the expression of lipid metabolism-related genes and proteins in both decidua and DSCs. In decidua, CD36 mRNA expression was significantly upregulated in HMD group compared to ND group, while expression levels of DGAT2, SREBP-1c, and SCD1 showed no significant changes (Fig. 5A). In DSCs, HHcy treatment significantly upregulated the mRNA expression of CD36, SREBP-1c, DGAT2, and SCD1 (Fig. 5B). At the protein level, CD36 and SREBP-1c expression were significantly increased in the decidua of HMD group (Fig. 5C). In DSCs, CD36 and SCD1 protein expression levels were elevated following HHcy treatment, while levels of SREBP-1c and DGAT2 remained unchanged (Fig. 5D).

These results suggest that CD36 may serve as a central mediator of HHcy-induced lipid metabolic dysregulation in decidua. Based on these findings, we hypothesized that HHcy suppresses VEGFA expression through CD36-mediated modulation of lipid metabolism. Moreover, given the observed enrichment of lipid metabolic pathways and prior evidence linking lipid metabolism to angiogenesis, we further speculated that this regulation might involve the peroxisome proliferator-activated receptor (PPAR) signaling pathway (Fig. 4C). Supporting this, Western blot analysis showed that HHcy stimulation significantly upregulated both PPARα and PPARγ expression in decidua and DSCs (Fig. 5E-F).

### HHcy inhibits VEGFA expression and secretion by upregulating CD36 expression in DSCs

To further validate the role of CD36 in mediating HHcy-induced effects, we treated DSCs with SSO, a specific CD36 inhibitor, and examined its impact on lipid metabolism and VEGFA expression.

**Fig. 6:**
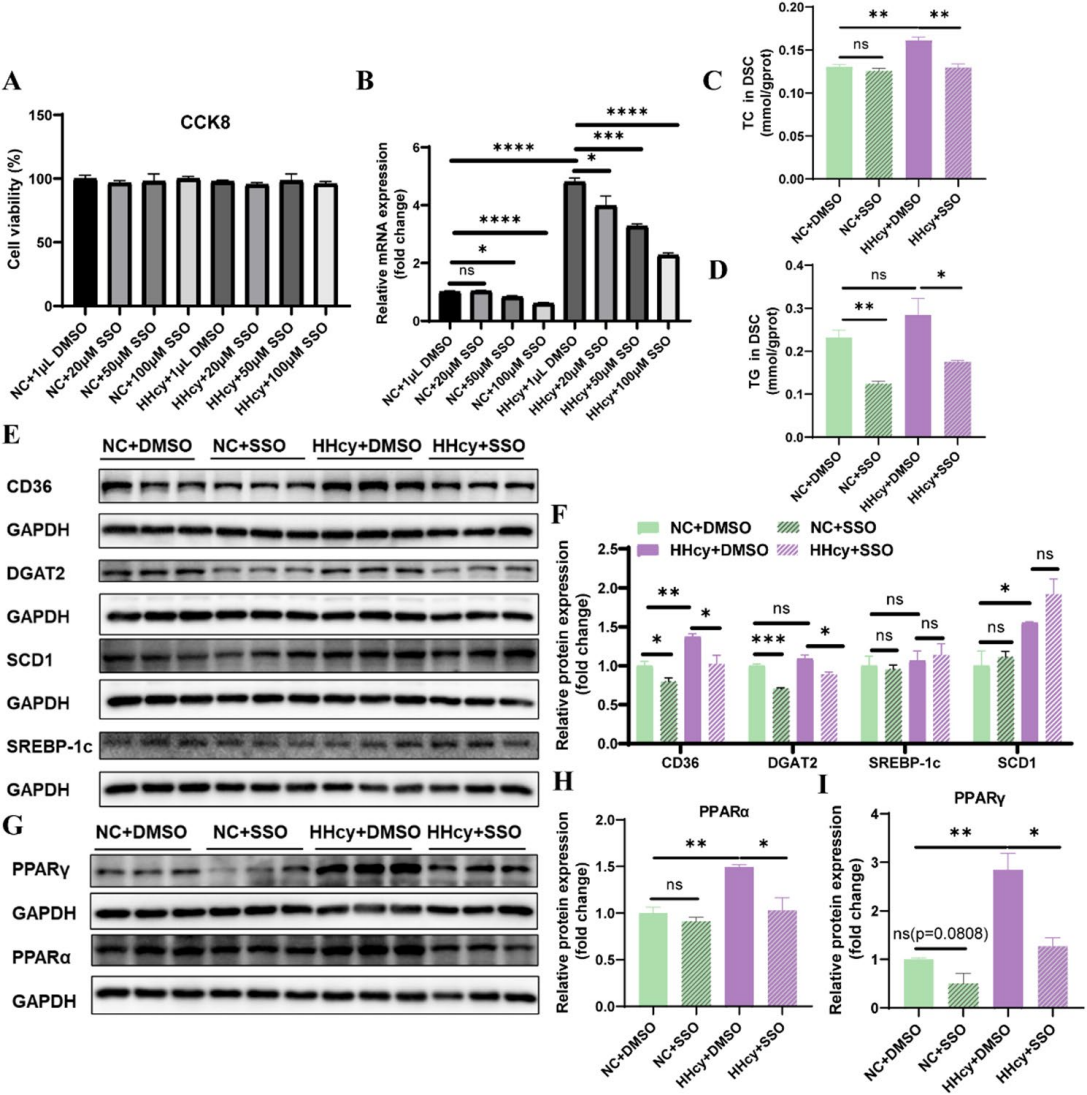
CD36 inhibitor can ameliorate HHcy-induced lipid metabolic disorder and upregulation of the PPAR signaling pathway. **A** Cell viability of DSCs after treatment with Hcy and/or CD36 inhibitor SSO for 24 h (*n* = 6 per group). **B** CD36 mRNA expression in DSCs after treatment with Hcy and/or SSO (*n* = 4 per group). **C** and **D** TC and TG levels in DSCs after treatment with Hcy and/or 100µM SSO (*n* = 3 per group). **E **and **F** The protein expression of CD36 and other lipid metabolism-related molecules in DSCs after treatment with Hcy and/or 100µM SSO (*n* = 3 per group). **G** and **H** The protein expression of PPARα and PPARγ in DSCs after treatment with Hcy and/or 100µM SSO (*n* = 3 per group). The data were expressed as mean ± SEM and statistical comparisons were assessed by unpaired two-tailed Student’s t-test. ns *P* > 0.05, * *P* < 0.05, ** *P* < 0.01, *** *P* < 0.001, **** *P* < 0.0001

CCK8 and RT-qPCR assays confirmed that 100 µM SSO effectively blocked the HHcy-induced upregulation of CD36 mRNA in DSCs without affecting cell viability (Fig. 6A and B). Western blot analysis corroborated that SSO reversed the HHcy-induced elevation in CD36 protein expression (Fig. 6E and F). As a result, 100 µM SSO was selected for subsequent experiments, with DMSO used as the vehicle control.

SSO treatment significantly reduced intracellular lipid accumulation induced by HHcy in DSCs (Fig. 6C and D). In parallel, Western blot results demonstrated that SSO downregulated CD36 and DGAT2 protein levels (Fig. 6E and F). Moreover, HHcy-induced upregulation of PPARα and PPARγ was attenuated by SSO, suggesting that CD36 inhibition modulates the PPAR signaling pathway (Fig. 6G–I).

**Fig. 7:**
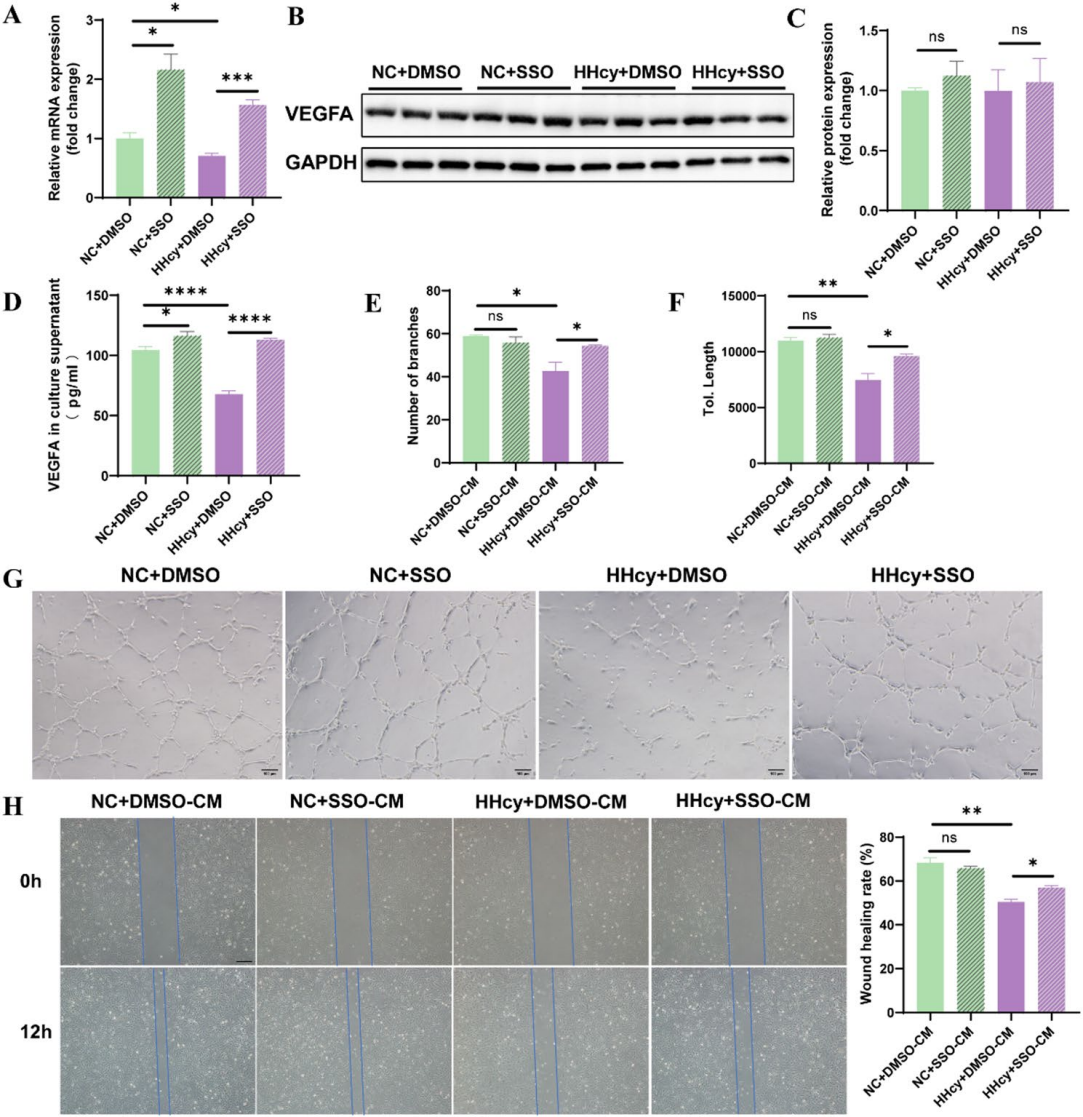
HHcy inhibits VEGFA expression and secretion by upregulating CD36 expression in DSCs, thereby impairing angiogenesis. **A** The VEGFA mRNA expression of DSCs after treatment with Hcy and/or 100µM SSO (*n* = 3 per group). **B** and **C** The VEGFA protein expression of DSCs after treatment with Hcy and/or 100µM SSO (*n* = 3 per group). **D** VEGFA concentration in the culture supernatant of DSCs after treatment with Hcy and/or 100µM SSO, measured by ELISA (*n* = 6 per group). **E–G** Conditioned medium (CM) collected from treated DSCs was used for HUVECs tube formation assays. Images were taken 6 h after incubation. Scale bar = 100 μm. Quantitative analysis of branch number and total tube length (*n* = 3 per group). **H** CM from treated DSCs was used for HUVECs wound healing assays. Images were taken at 0 h and 12 h. Scale bar = 50 μm. The wound healing rate was quantified (*n* = 3 per group). The data were expressed as mean ± SEM and statistical comparisons were assessed by unpaired two-tailed Student’s t-test. ns *P* > 0.05, * *P* < 0.05, ** *P* < 0.01, *** *P* < 0.001, **** *P* < 0.0001

RT-qPCR analysis revealed that SSO treatment alone (NC + SSO) elevated VEGFA mRNA expression in DSCs compared to the vehicle control (NC + DMSO), and co-treatment with Hcy and SSO (Hcy + SSO) significantly restored VEGFA mRNA levels suppressed by HHcy (Hcy + DMSO) (Fig. 7A). Although intracellular VEGFA protein levels remained unchanged across groups (Fig. 7B-C), ELISA analysis showed that VEGFA secretion into the culture medium was modestly increased by SSO alone and significantly restored in the Hcy + SSO group compared to the Hcy + DMSO group (Fig. 7D).

To assess the functional impact of VEGFA secretion, we performed HUVECs tube formation and wound healing assays using CM from the treated DSCs. CM from NC + SSO-treated DSCs (NC + SSO-CM) had no notable effect on HUVECs tube formation compared to NC + DMSO-CM (Fig. 7E–G). However, CM from Hcy + SSO group (Hcy + SSO-CM) significantly improved tube length and branching compared to Hcy + DMSO-CM group (Fig. 7E–G). Likewise, in the wound healing assay, CM from Hcy + SSO-treated DSCs restored the impaired migratory capacity of HUVECs observed in HHcy group, while NC + SSO-CM had no effect (Fig. 7H).

Collectively, these results indicate that HHcy disrupts lipid metabolism in decidua by upregulating CD36, which in turn suppresses VEGFA expression and secretion in DSCs via the PPAR signaling pathway, ultimately impairing angiogenesis. Pharmacological inhibition of CD36 by SSO partially rescues these effects, identifying CD36 as a potential therapeutic target for HHcy-induced angiogenic dysfunction.

## Discussion

Maternal HHcy has a significant impact on fetal growth, but its underlying mechanisms remain poorly defined [26]. In this study, we investigated the effects of HHcy on fetal development and maternal-fetal interface angiogenesis. Mechanistically, we propose that HHcy disrupts decidual lipid metabolism by upregulating CD36 expression in DSCs, thereby suppressing VEGFA expression and secretion, potentially through activation of the PPAR signaling pathway. This cascade ultimately impairs angiogenesis. To our knowledge, this is the first study to elucidate the role of lipid metabolism in regulating decidual angiogenesis, and it provides new insights into the underlying mechanisms involved.

Previous studies have linked HHcy to FGR across multiple experimental models. For instance, exposure of chicken embryos to Hcy during the critical window of vascular development significantly reduced their survival in vitro, and stimulation of mouse embryos with Hcy during neural tube closure induced marked growth retardation [21, 27]. Similarly, HHcy induced by high-methionine diets in pregnant Wistar rats resulted in reduced fetal birth weight [28]. In humans, numerous clinical studies have also demonstrated that maternal HHcy is closely associated with an elevated risk of IUGR [10–13]. Consistent with these findings, our study demonstrated that HHcy impaired early embryonic development and restricted fetal growth beginning in mid-gestation, with effects persisting into late pregnancy. Preliminary analysis of placental phenotypes suggests that HHcy alters both placental weight and structure. Given that nutrient exchange between mother and fetus relies predominantly on the placenta during mid and late gestation, these placental alterations may underlie the HHcy-induced FGR [29].

The decidua in early pregnancy and the placenta in later stages, comprising the maternal-fetal interface, are central to fetal development, where proper angiogenesis is critical for fetal development [14, 15]. Our findings revealed reduced vascular density in both decidua and placenta across gestation in HHcy-exposed rats. During early pregnancy, HHcy downregulated VEGFA expression while upregulating PLGF expression in the decidua. Previous studies have shown similar findings: in the HHcy model induced by folate deficiency, VEGFA and PLGF expression in the decidua were reduced during early pregnancy [30]. VEGFA is a dominant regulator of early decidual angiogenesis, whereas increased PLGF may reflect a compensatory mechanism [16, 17]. Such imbalance in angiogenic signaling may disrupt the transition from branching to non-branching angiogenesis, impairing placental vascular development [16, 17].

Our findings indicate that maternal HHcy induces lipid accumulation in the decidua and reduces VEGFA secretion, whereas alleviating lipid deposition enhances VEGFA secretion, suggesting a regulatory role of lipid metabolism in decidual angiogenesis. Mechanistically, several pathways may be involved: lipid metabolites serve as signaling molecules that regulate nuclear receptors like PPARs, which in turn influence VEGFA expression; lipid accumulation may elevate oxidative stress in DSCs, negatively affecting VEGFA synthesis and release; lipid metabolism and mitochondrial function are interlinked, and mitochondrial dysfunction due to lipid dysregulation may alter the cellular energy state and metabolic signaling in DSCs, thus impairing VEGFA production; metabolic reprogramming of the decidua during pregnancy-essential for embryonic development-includes shifts in lipid metabolism, which may influence VEGFA expression [31–34]. By analyzing the RNA-sequencing data from decidual tissues, we found that the PPAR signaling pathway exhibited a trend toward enrichment in HHcy rats, suggesting that lipid metabolism may regulate decidual angiogenesis through PPAR signaling in our model. Notably, in corneal tissue, endothelial cells, and prostate cancer cell lines, activation of the PPAR pathway has been reported to inhibit angiogenesis by suppressing VEGFA expression [35–37].

Our data show that pharmacological inhibition of CD36 improved the lipid metabolism disorder caused by HHcy, which identified CD36 as a key mediator linking HHcy to lipid metabolic disruption in DSCs. Elevated serum Hcy levels have been associated with hypertriglyceridemia, hypercholesterolemia, and increased LDL cholesterol in the Chinese population [38]. Moreover, HHcy induced by HMD exacerbates lipid metabolism disturbances and promotes lipid plaque formation in mice [39]. CD36, a membrane glycoprotein, facilitates the uptake of long-chain fatty acids and plays a pivotal role in lipid metabolism regulation [40]. The relationship between CD36 and lipid dysregulation has been documented in various human and animal studies [41]. CD36 expression is elevated in the livers of mice and patients with metabolic-associated fatty liver disease, while hepatocyte-specific CD36 knockout alleviates hepatic steatosis and insulin resistance [42]. Moreover, CD36 has further been reported to mediate lipid metabolism crosstalk between DSCs and decidual macrophages [43]. However, no prior studies have investigated the role of CD36 in regulating VEGFA at the maternal-fetal interface.

Despite these insights, several limitations warrant mention. First, we did not evaluate the roles of other methionine cycle intermediates, such as the SAM/SAH ratio which is an indicator of methylation ability, and its changes may lead to interference in epigenetic reprogramming during pregnancy, resulting in defects in embryonic and placental development. Second, the effect of HHcy on other decidual cell types (e.g., trophoblasts, NK cells, macrophages, and endothelial cells) remains unexplored. Finally, although CD36 inhibition reversed key phenotypes in vitro, validation in vivo-such as using decidua-specific CD36 knockout models-would strengthen the therapeutic relevance, and the use of PPAR inhibitors or agonists would provide complementary mechanistic evidence and enhance the overall robustness of the conclusion. Also, our study focused only on male fetuses; sex-specific responses should be investigated in future studies.

**Fig. 8:**
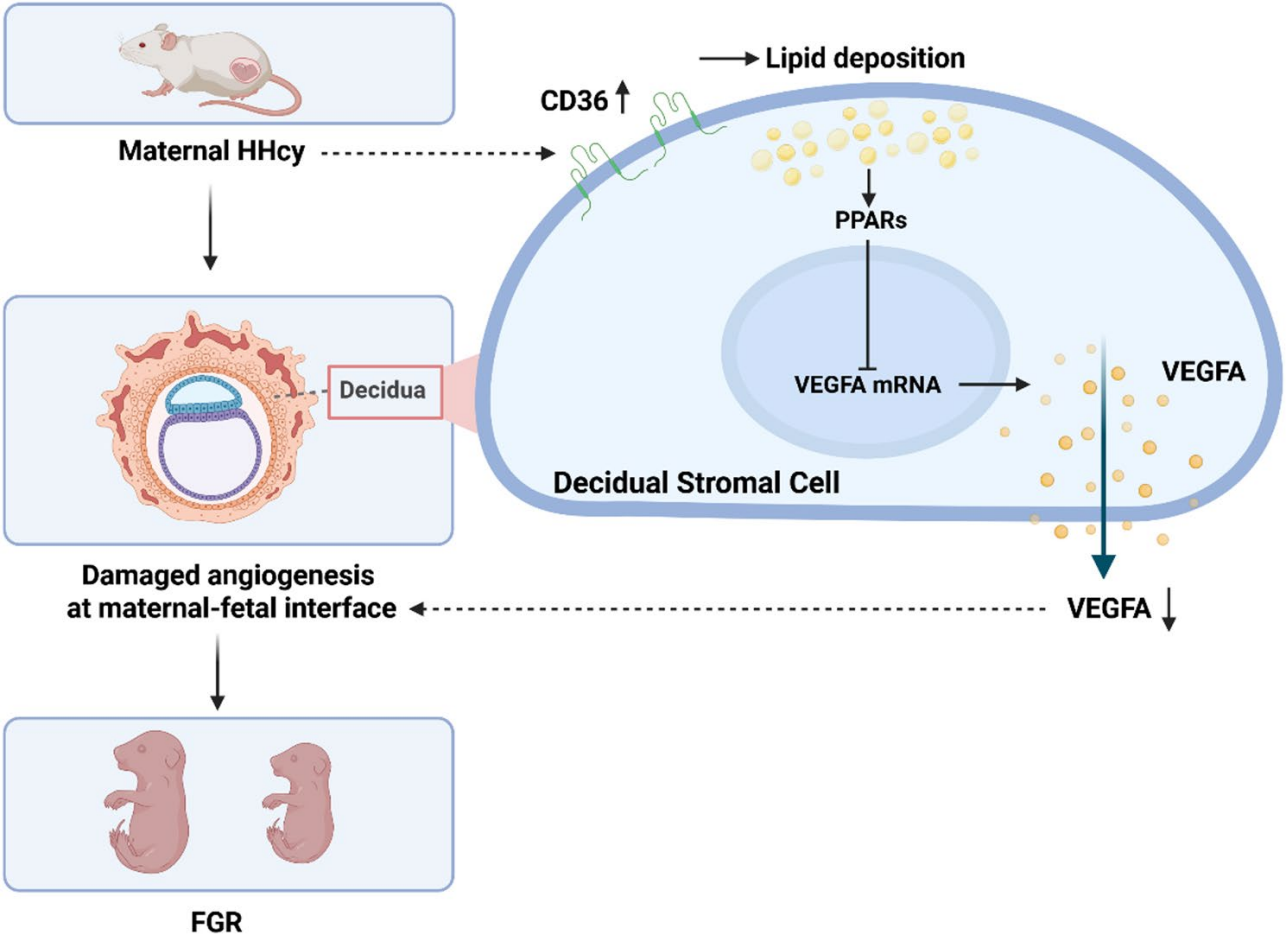
Schematic diagram of this study. HHcy leads to lipid deposition in decidua by upregulating CD36 expression in DSCs, which suppresses VEGFA expression and secretion possibly by activating the PPAR signaling pathway, thereby impairing angiogenesis at the maternal-fetal interface and causing FGR. The diagram was created at https://BioRender.com

In conclusion, our findings reveal a novel mechanism by which HHcy contributes to FGR via impaired angiogenesis at the maternal-fetal interface. We identified CD36-mediated lipid dysregulation in DSCs as upstream regulators of VEGFA suppression and angiogenic dysfunction (Fig. 8). This is the first report to demonstrate the regulatory role of lipid metabolism in decidual angiogenesis and to propose potential mechanisms underlying this process. These insights provide a mechanistic link between HHcy, lipid metabolism, and impaired angiogenesis at the maternal-fetal interface, and position CD36 as a potential therapeutic target for HHcy-related pregnancy complications.

## Supplementary Information

Below is the link to the electronic supplementary material.

Supplementary Material 1

Supplementary Material 2

## Data Availability

All data are available in the article or Supplementary Material. All other data supporting the findings of this study are available from the corresponding author upon reasonable request.

## Abbreviations

CCK8: Cell counting kit-8
CM: Conditioned medium
DEGs: Differentially expressed genes
DSCs: Decidual stromal cells
FGR: Fetal growth retardation
Hcy: Homocysteine
HHcy: Hyperhomocysteinemia
HMD: High methionine diet
HUVECs: Human umbilical vein endothelial cells
Met: Methionine
ND: Normal diet
PLGF: Placental growth factor
SAM: S-adenosylmethionine
SAH: S-adenosyl-L-homocysteine
SSO: Sulfo-N-succinimidyl oleate sodium
TC: Total cholesterol
TG: Triglycerides
VEGFA: Vascular endothelial growth factor A
