# Supplementary material for: Maternal hyperhomocysteinemia induces fetal growth restriction by suppressing angiogenesis at the maternal-fetal interface
Source: Cell Biosci. 2026 Jan 9;16:17. doi: 10.1186/s13578-025-01529-0 (PMC12882372; doi:10.1186/s13578-025-01529-0)
Supplement: Supplementary file 1 — Supplementary Material 1 [file 13578_2025_1529_MOESM1_ESM.docx]

| Antibody name | Supplier | Catalog number | Dilution |
| --- | --- | --- | --- |
| Anti-VEGFA antibody | Abcam, England | ab214424 | 1:1000 |
| PLGF Rabbit pAb | Abclonal, China | A1727 | 1:1000 |
| HIF1α Rabbit mAb | CST, USA | 14179 | 1:1000 |
| Anti-CA9 antibody | Abcam, England | ab243660 | 1:1000 |
| Vinculin Rabbit mAb | Abclonal, China | A2752 | 1:1000 |
| GAPDH Monoclonal antibody | Proteintech, China | 60004-1-Ig | 1:10000 |
| CD36 Polyclonal antibody | Proteintech, China | 18836-1-AP | 1:1000 |
| Anti-SREBP-1c antibody | Abcam, England | ab18481 | 1:1000 |
| DGAT2 Polyclonal antibody | Proteintech, China | 17100-1-AP | 1:1000 |
| Anti-SCD1 antibody | Abcam, England | ab236868 | 1:1000 |
| Anti-PPARα antibody | Abcam, England | Ab24509 | 1:1000 |
| PPARγ Monoclonal antibody | Proteintech, China | 66936-1-Ig | 1:5000 |

Supplementary Table S1. The primary antibodies used for western blot in this study.

Supplementary Table S2. RT-qPCR primers used in this study.

| Gene | Primer sequence |
| --- | --- |
| *VEGFA* | F: CGGACAGACAGACAGACACC |
|  | R: AGCCCAGAAGTTGGACGAAA |
| *PLGF* | F: GCTGTTCCCTTGCTTCCTGC |
|  | R: CCCTTGGGTCTCCTCCTTTCC |
| *HIF1α* | F: TCACAAATCAGCACCAAGCAC |
|  | R: AAGGGGAAAGAACAAAACACG |
| *CA9* | F: GCTCCATACGCTCTCCGTTT |
|  | R: GGCTAGGATGTCACCAGCAG |
| *GAPDH* | F: ATGGGAAGCTGGTCATCAAC |
|  | R: GGATGCAGGGATGATGTTCT |
| *CD36* | F: GCTGTTCCCTTGCTTCCTGC |
|  | R: CCCTTGGGTCTCCTCCTTTCC |
| *DGAT2* | F: TCCTTCCTGGTGCTAGGAGT |
|  | R: CTCGAAAATAGCGCCACACG |
| *SREBP-1c* | F: CTTCAGTCTTGGCTCGCCTC |
|  | R: GTACCCACTGGCCTTCTCAC |
| *SCD1* | F: ACACCTTGCTCTGGGGGATA |
|  | R: CTCCACAGGCGATGAGCC |
