# Supplementary figures and images for: Maternal hyperhomocysteinemia induces fetal growth restriction by suppressing angiogenesis at the maternal-fetal interface
Source: Cell Biosci. 2026 Jan 9;16:17. doi: 10.1186/s13578-025-01529-0 (PMC12882372; doi:10.1186/s13578-025-01529-0)

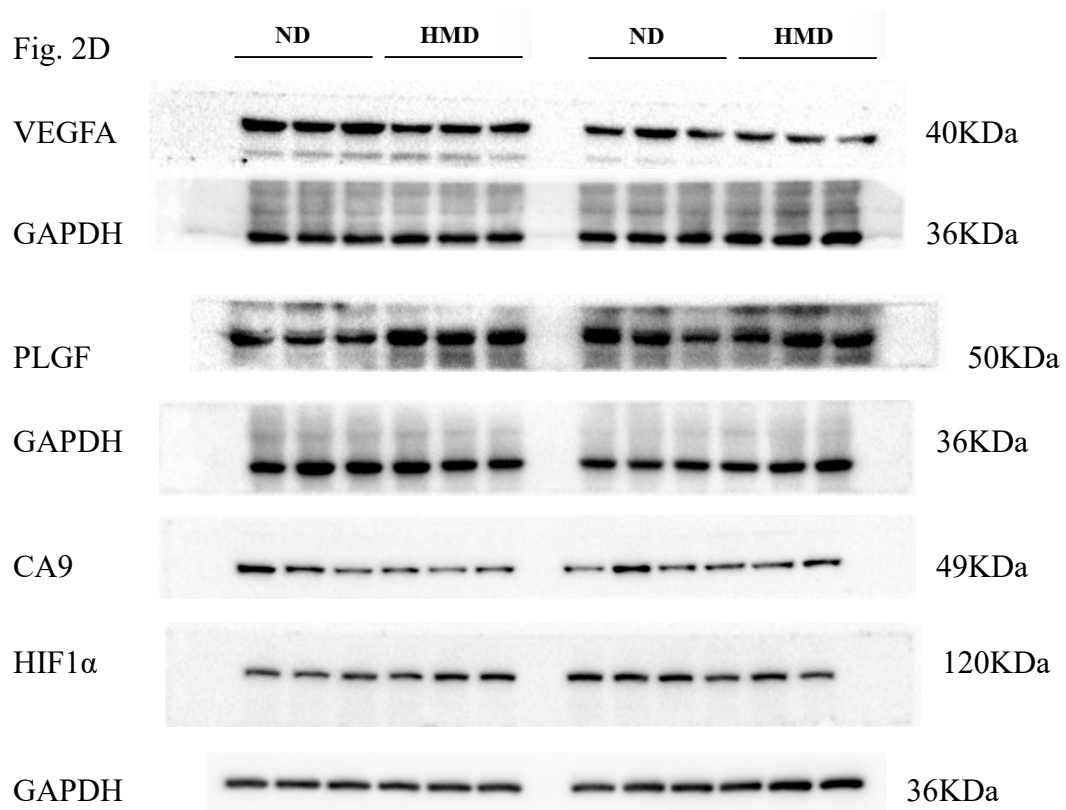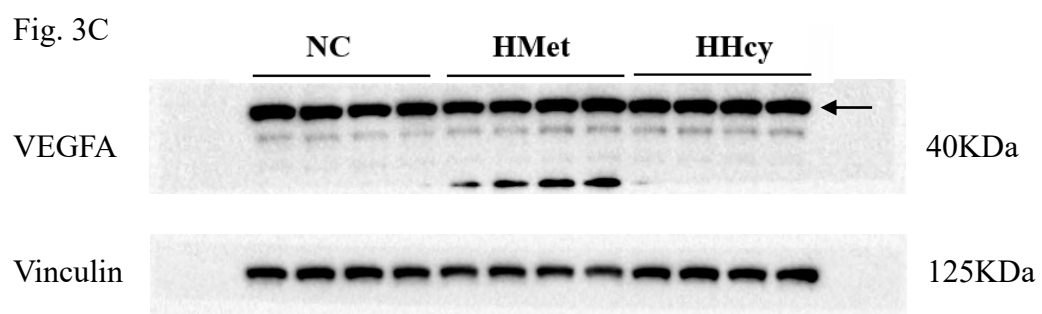

Fig. 5C

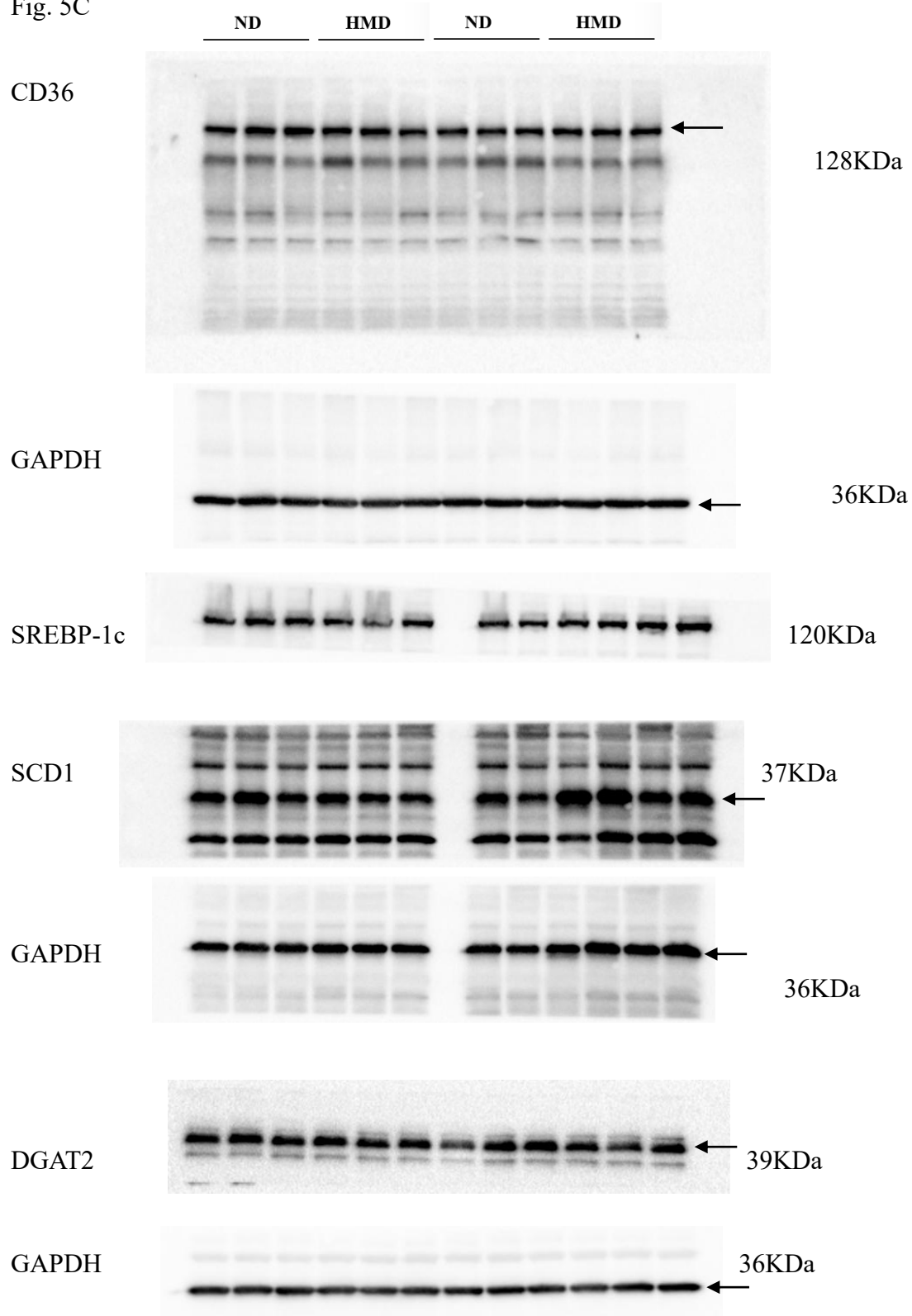

Fig. 5D

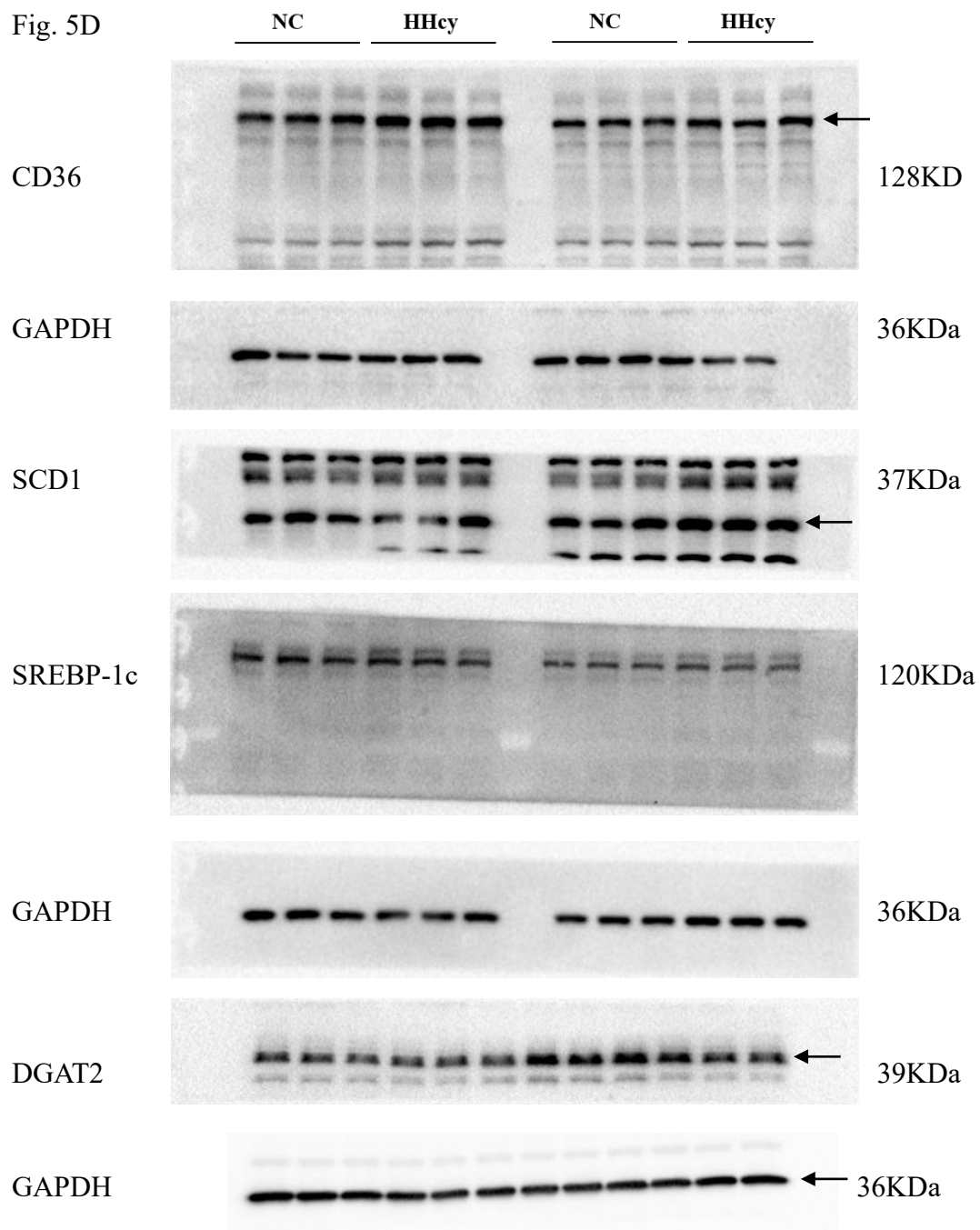

Fig. 5E

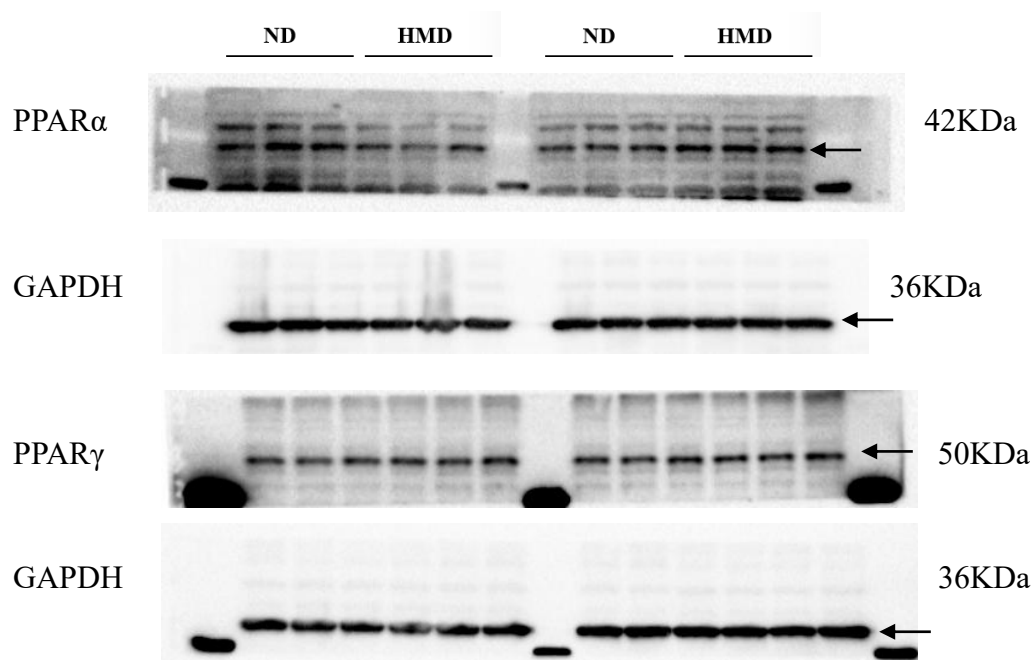

Fig. 5F

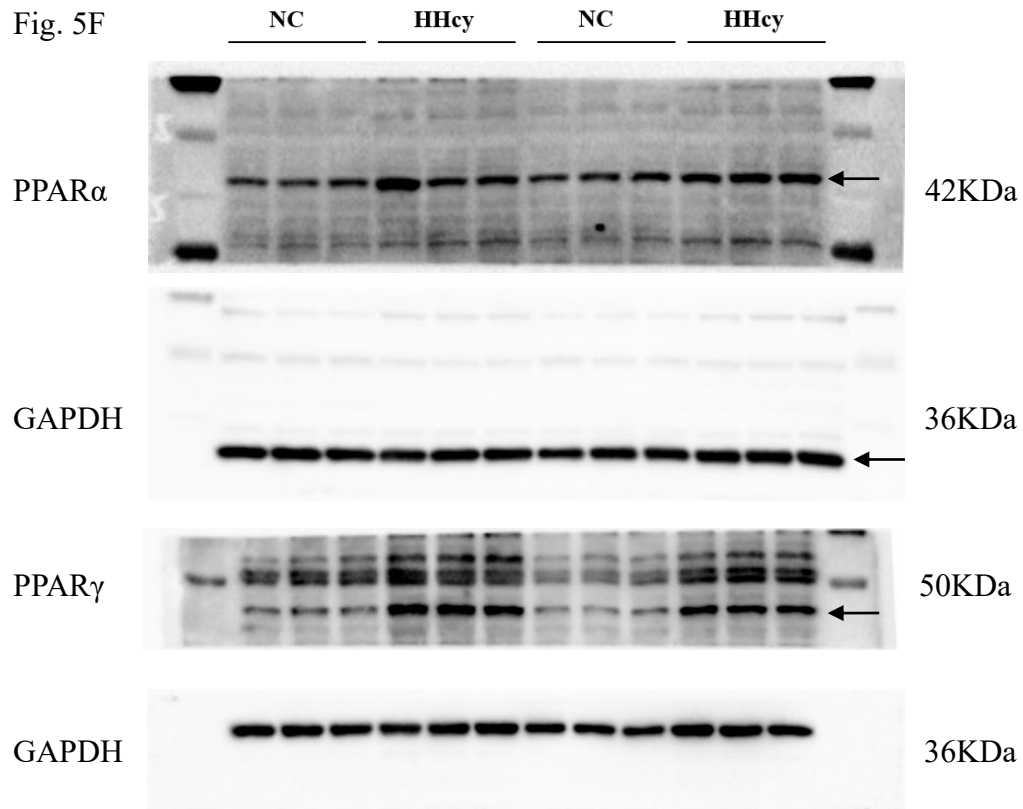

Fig. 6E

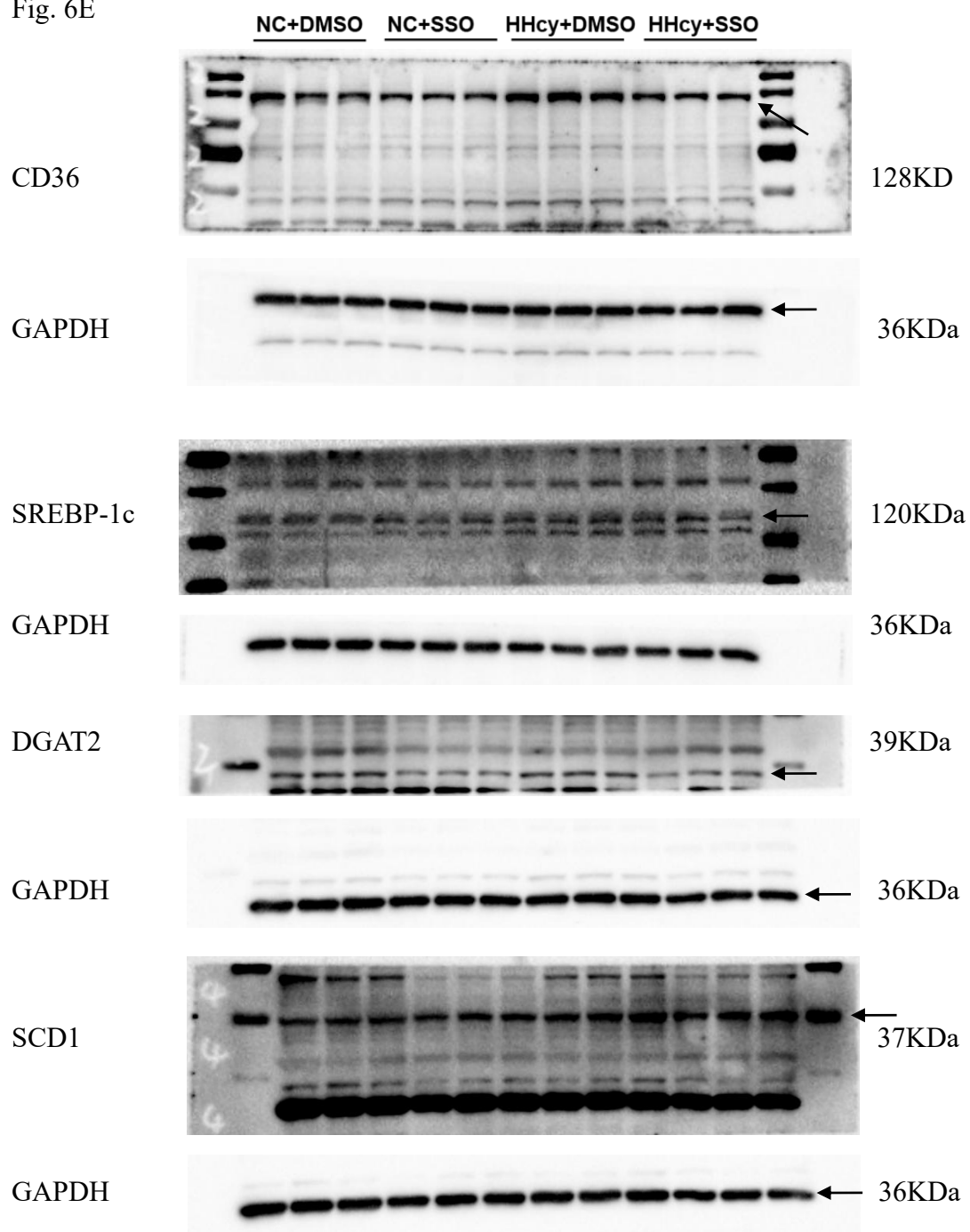

Fig. 6G

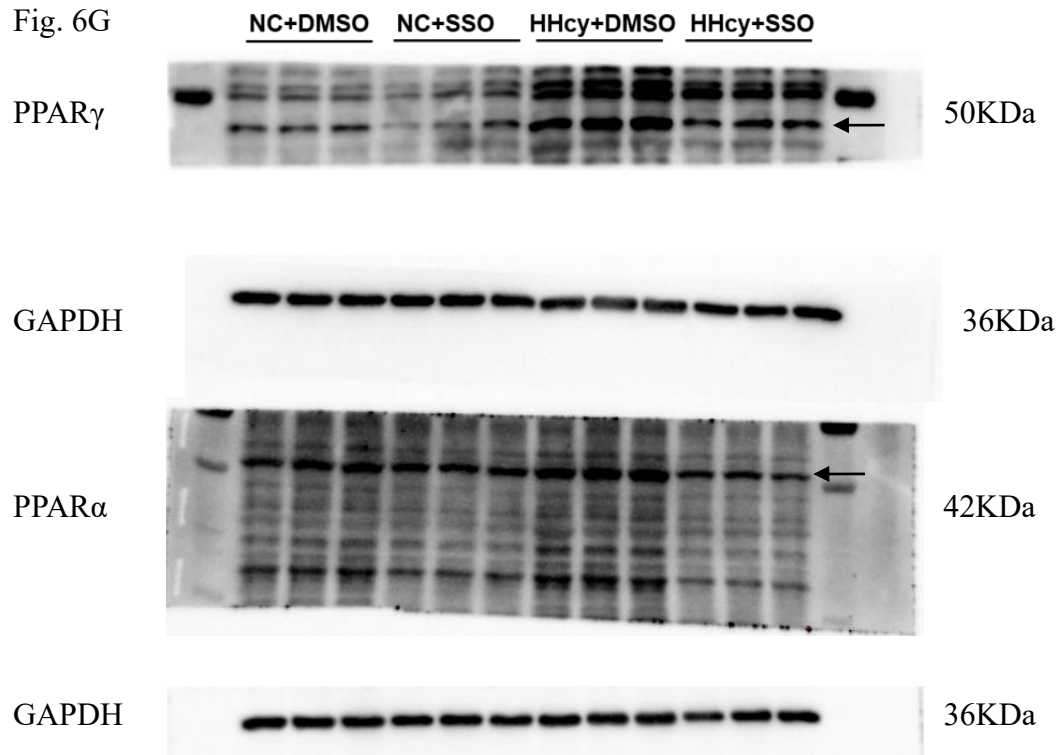

Fig. 7B

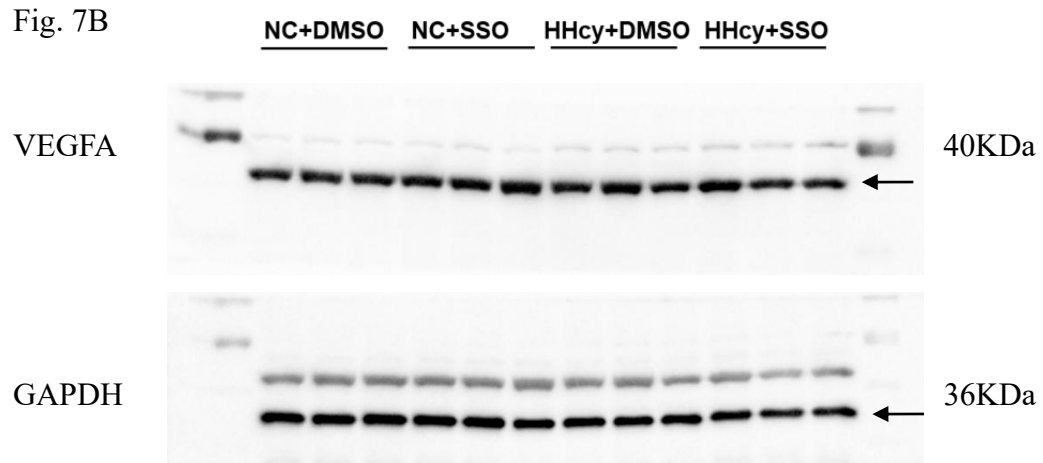

Supplement: Supplementary file 2 — Supplementary Material 2 [file 13578_2025_1529_MOESM2_ESM.pdf]
